# An inhibitory role of Arg-84 in anion channelrhodopsin-2 expressed in *Escherichia coli*

**DOI:** 10.1038/srep41879

**Published:** 2017-02-02

**Authors:** Satoko Doi, Takashi Tsukamoto, Susumu Yoshizawa, Yuki Sudo

**Affiliations:** 1 Graduate School of Medicine, Dentistry and Pharmaceutical Sciences, Okayama University, Okayama 700-8530, Japan; 2 Atmosphere and Ocean Research Institute, The University of Tokyo, Chiba 277-8564 Japan

## Abstract

Anion channelrhodopsin-2 (ACR2) was recently identified from the cryptophyte algae *Guillardia theta* and has become a focus of interest in part because of its novel light-gated anion channel activity and its extremely high neural silencing activity. In this study, we tried to express ACR2 in *Escherichia coli* cells as a recombinant protein. The *E. coli* cells expressing ACR2 showed an increase in pH upon blue-light illumination in the presence of monovalent anions and the protonophore carbonyl cyanide *m*-chlorophenylhydrazone (CCCP), indicating an inward anion channel activity. Then, taking advantage of the *E. coli* expression system, we performed alanine-scanning mutagenesis on conserved basic amino acid residues. One of them, R84A, showed strong signals compared with the wild-type, indicating an inhibitory role of R84 on Cl^−^ transportation. The signal was strongly enhanced in R84E, whereas R84K was less effective than the wild-type (i.e., R84). These results suggest that the positive charge at position 84 is critical for the inhibition. Thus we succeeded in functional expression of ACR2 in *E. coli* and found the inhibitory role of R84 during the anion transportation.

In nature, a wide variety of photoreceptive proteins play critical roles in organisms as receptors of sunlight containing both harmful and useful wavelengths of light. Because proteins consisting of amino acid residues don’t absorb visible light, a chromophore molecule showing absorption in the visible region is required for photoreception. A member of the 7 transmembrane (TM) domain family of proteins, rhodopsins have vitamin-A aldehyde retinal as their chromophore, in which a specific lysine residue located in the middle of the TM region binds to a retinal molecule resulting in formation of the protonated Schiff base linkage^1^,^2^,^3^. The photoabsorption of the retinal in rhodopsins triggers the stepwise reactions that accompany the *trans-cis* photoisomerization upon formation of the early photointermediate^3^,^4^. Rhodopsin molecules are roughly classified into two groups, microbial type-1 rhodopsins and animal type-2 rhodopsins^1^,^5^. The type-1 rhodopsins are widely distributed in a variety of organisms including halophilic archaea, proteobacteria, cyanobacteria, fungi and algae^1^,^5^. Such variation mirrors the diversity in biological function^1^,^5^,^6^. In 1971, Oesterhelt and Stoeckenius identified the first type-1 rhodopsin from the archaeon *Halobacterium salinarum* (formerly halobium)^7^. They named that protein bacteriorhodopsin (BR) and determined its biological function as a light-driven proton pump to produce adenosine triphosphate (ATP). In the 45 years since then, BR has become a model for the simplest and most essential features necessary in an active ion transporter^3^. New discoveries started at the end of the last century when techniques in mass genome sequencing and bioinformatics started to advance dramatically^8^. As an example, a light-driven Na^+^ pumping rhodopsin named KR2 was discovered in 2013^9^. Eventually many studies have revealed that type-1 rhodopsins exist not only in archaea but also in eukaryotes and eubacteria, indicating the biological importance of type-1 rhodopsins in nature^3^,^10^.

During the extensive genomic studies, two novel rhodopsins, channelrhodopsin-1 (ChR1) and channelrhodopsin-2 (ChR2), that account for the phototactic behaviors of the eukaryotic algae *Chlamydomonas reinhardtii*, were identified in 2002 and 2003^11^,^12^,^13^, respectively. Both rhodopsins absorb blue light (ca. 470~500 nm) and function as light-gated cation-selective ion channels, but they differ in their saturating light intensity similar to the difference between rod and cone visual pigments. ChR1 and ChR2 can conduct Na^+^, K^+^ and Ca^2+^ at a similar rate in addition to H^+^. Thus, organisms expressing ion pumping rhodopsins and ion channeling rhodopsins maintain ion concentrations in the cell by light. It is well-known that all organisms control their activities by changing the ion gradient between the outside and the inside of cell membranes. Therefore, the biological activities of organisms, such as neural activities, can be controlled using rhodopsins with visible light in high temporal and spatial resolution. In fact, ion pumping rhodopsins, such as Archaerhodopsin-3 (AR3), *Natronomonas pharaonis* Halorhodopsin (NpHR) and their color variants, have been utilized as neural silencers in mammalian neurons^14^,^15^,^16^, while by taking advantage of ChR2 producing a long half-life current, ChR2 has been utilized as a neural activator^17^. The new technology to control neurons is called “Optogenetics”^18^. Thus, rhodopsins have become a focus of interest in part because of their importance to the general understanding of ion transportation through integral membrane proteins and to optimize them for optogenetics^19^. On the basis of this background, many research groups have investigated the structure-function relationship of rhodopsins in this decade. Regarding ChRs, the cyclic photoreaction of ChR2^20^ and the ion transport pathway of ChRs^21^ were extensively investigated using yeast, insect, and animal cells as hosts. Because the *Escherichia coli* expression system has great advantages with respect to its growth rate and ease of genetic/proteomic modifications, in comparison with animal systems, many researchers have tried to express ChRs in *E. coli*. However, no report on the functional expression of ChRs in *E. coli* has been published. In fact, we successfully expressed a ChR1 variant lacking both the N- and C-termini in *E. coli* as a holoprotein, however it did not show the light-gated ion channel activity^22^.

In 2015, a novel type of ChR-like proteins, anion ChRs (ACRs), was discovered in the cryptophyte algae *Guillardia theta* CCMP2712 and was shown to function as a light-gated anion channel^23^. It has been revealed that ACRs absorb blue~green light (470~520 nm) and transport monovalent anions such as Cl^−^ and Br^−^ during the cyclic reaction called the photocycle^23^. In addition to their functional novelty, one type of ACR, anion ChR-2 (ACR2), showed an extremely high performance (ca. 1000-fold) as an optogenetic tool for neural silencing in comparison with a ChR-based artificial ACR (slow ChloC) and the proton pumping rhodopsin AR3^23^. In this study, we tried to express ACR2 in *E. coli* cells and then, using the expression system, we performed mutational analysis on conserved basic amino acid residues to investigate the anion transport mechanism.

## Results and Discussion

### Functional expression of ACR2 in *E. coli* cells

**Figure 1:**
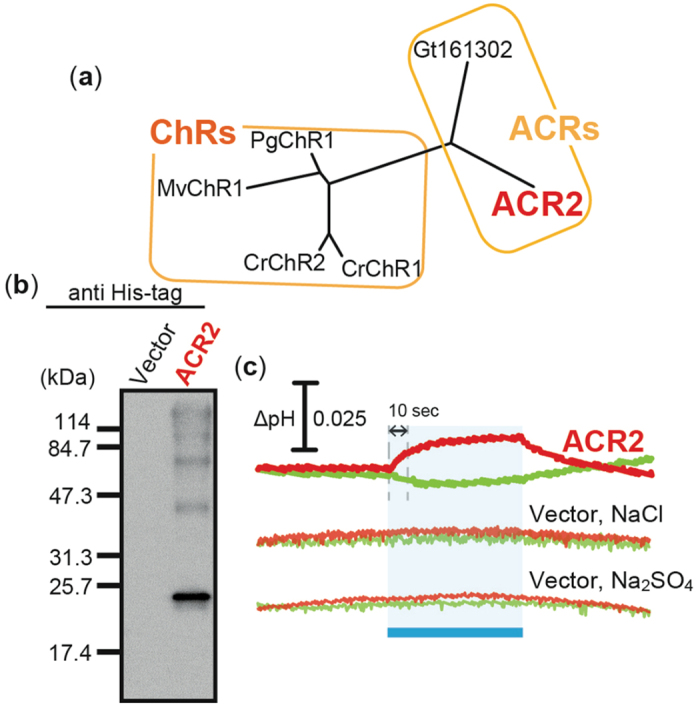
Expression of ACR2 in *E. coli* cells. (**a**) Phylogenetic tree of ChRs and ACRs from alga. CrChR1 (Genbank accession number, AF508965), CrChR2 (AF508966), MvChR1 (JF922293), PgChR1 (JQ241366), ACR2 (EKX35002) and Gt161302 (EKX52099) indicate ChR1 from *Chlamydomonas reinhardtii*, ChR2 from *C. reinhardtii*, ChR1 from *Mesostigma viride*, ChR1 from *Pyramimonas gelidicola*, ACR2 from *Guillardia theta*, and ACR-like protein from *G. theta*, respectively. (**b**) Detection of protein expression. ACR2 was expressed in *E. coli* BL21(DE3) cells with a His-tag at the C-terminus. The protein was detected using an anti His-tag antibody. Cells harboring the pET22b vector plasmid alone were used as a negative control. (**c**) Light-induced pH changes. Cells were suspended in 150 mM NaCl or 100 mM Na_2_SO_4_. These cells were illuminated with blue light (480 ± 10 nm, ca. 9 mW/cm^2^) for 3 min (blue band). The red and green lines indicate pH changes in the presence or absence of 10 μM CCCP, respectively. The initial slope amplitude of the light-induced pH change from 0 to 10 sec upon illumination was used to evaluate the ion transport activities of ACR2.

To date, no functional expression of ChRs in *E. coli* cells has been achieved. Recently, several ChR-like proteins and ACRs have been discovered in eukaryotic green alga. Among them, in this study, we focused on 4 proteins, MvChR1, PgChR1, ACR2 and Gt161302 (Fig. 1a), and prepared expression plasmids encoding their 7 TM domains. A 6-histidine tag was inserted at each C-terminus to enable detection of apoproteins. Figure 1b shows western blot analysis of ACR2 using an anti His-tag antibody. A strong positive signal appeared for ACR2 around the marker band of 25.7 kDa, which matches approximately the expected value (30.8 kDa), indicating the successful expression of ACR2 in *E. coli* cells as a recombinant apoprotein. On the other hand, almost no bands were observed for MvChR1, PgChR1 or Gt161302 (data not shown), indicating little or no expression of those apoproteins in *E. coli* cells. Presumably, transcription and/or translation did not work well for MvChR1, PgChR1 and Gt161302, although the codon usage was optimized for the *E. coli* translation system. Another possibility is that the His-tag was digested during translation and/or translocation due to the flexibility of the C-terminus. To verify whether those rhodopsins show any light-gated ion transport activity, we measured the light-induced pH change of *E. coli* cells harboring expression plasmids encoding MvChR1, PgChR1, ACR2 and Gt161302. The cell suspensions were washed in 150 mM NaCl solution and were illuminated with blue light (ca. 480 nm) for 3 min. Almost no signal was observed for MvChR1, PgChR1 or Gt161302 (data not shown), indicating their non-functional expression in *E. coli* cells, while a weak decrease and a robust increase in pH were observed for ACR2 in the absence (green line) or presence (red line) of 10 μM of the protonophore CCCP, respectively, upon illumination (Fig. 1c). The altered pH recovered to the original pH within a few minutes after turning off the light. The pH gradient between the inside and the outside of the cell membrane disappeared before the illumination in the presence of CCCP, because CCCP is a proton-selective ionophore. Therefore, the inward H^+^ movement (increase in pH) in the presence of CCCP (red line) is equivalent to the transportation of some anions or cations with a subsequent secondary H^+^ movement across the membrane. Thus, ACR2 expressed in *E. coli* cells is considered to be a light-gated outward sodium (Na^+^) or inward chloride (Cl^−^) transporter. This is consistent with the results reported so far that ACR2 works as a light-gated anion channel when it is heterologously expressed in HEK293 cells^23^.

On the other hand, a weak but significant decrease in pH was observed for the cell suspension without CCCP (green line in Fig. 1c), suggesting the possibility that ACR2 also has an outward H^+^ pumping activity. This is because, in this experimental condition, the proton gradient exists from the outside to the inside of the cells (pH values of outside and inside are ca. 6.4 and ca. 7.0, respectively) and therefore protons should be transported from outside to inside if ACR2 is a proton channel. These results suggest that, by addition of the protonophore CCCP, the outward H^+^ movement by the H^+^ pumping activity was impaired and the secondary inward H^+^ movement by outward sodium (Na^+^) or inward chloride (Cl^−^) transport activity of ACR2 was enhanced. Thus ACR2 is assumed to be a light-dependent Cl^−^ (or Na^+^)/H^+^ antiporter (or symporter). However the decrease in pH is in contradiction with firmly established properties of ACRs obtained in patch-clamp experiments^23^. Therefore the negative signal may reflect secondary processes such as control of respiration, opening of native ion channels and activation of other ion transport systems. Further studies will be needed to elucidate more precise mechanism of the proton transportation.

**Figure 2:**
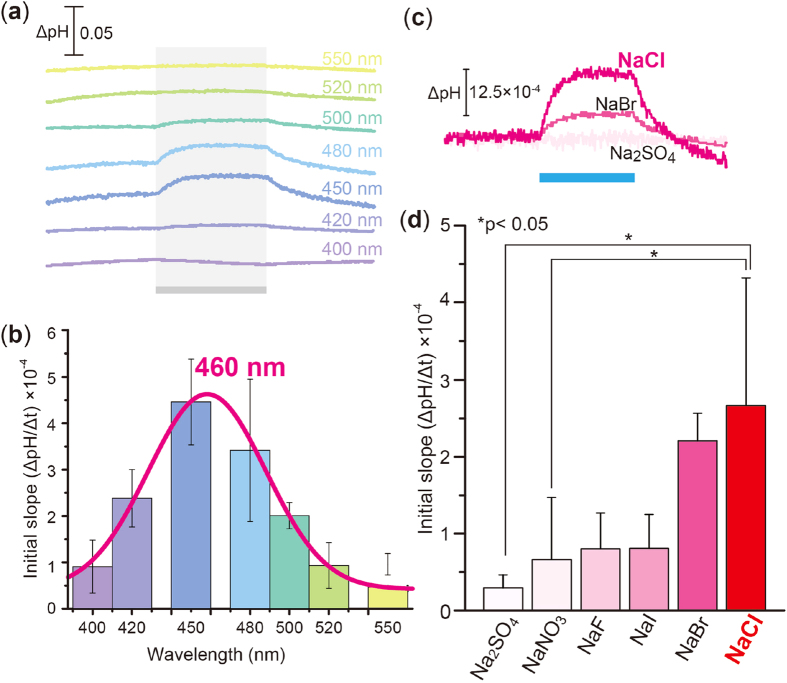
Ion transport function of ACR2 in *E. coli* cells. (**a**) Light-induced pH changes at varying wavelengths of visible light. *E. coli* cells were suspended in 300 mM NaCl solution, and were illuminated for 3 min (gray bands) with varying wavelengths of light (400, 420, 450, 480, 500, 520 and 550 ± 10 nm, 8–10 mW/cm^2^) in the presence of 10 μM CCCP. (**b**) The action spectrum. The initial slope amplitude was normalized by the light intensity and was plotted against the wavelengths of light. Several independent experiments were averaged (n = 3–12). Error bars represent standard deviations. Data were fitted by a normal Gaussian distribution (red line). (**c**) Light-induced pH changes of *E. coli* cell suspensions expressing ACR2 in solutions containing 100 mM Na_2_SO_4_, 300 mM NaCl, NaBr, NaI, NaF or NaNO_3_. The suspensions were illuminated with blue light (480 ± 10 nm, ca. 9 mW/cm^2^) for 3 min in the presence of 10 μM CCCP. (**d**) Substrate dependency of ion transport activities of ACR2. Several independent experiments were averaged (n = 3–18). The error bars represent the standard deviation. Statistical significance was evaluated by the Mann-Whitney test for two comparisons.

The robust increase in pH upon illumination allowed us to measure the action spectrum. As shown in Fig. 2a, we measured the light-induced pH change at varying wavelengths of light in a solution containing 300 mM NaCl. The initial slope amplitude of the light-induced pH change from 0 to 10 sec upon illumination (see Fig. 1c) was obtained in more than 3 independent experiments and was plotted against the wavelengths of light (Fig. 2b). From those data, the absorption maximum of ACR2 is roughly estimated to be 460 nm, which is close to that of ACR2 expressed in HEK293 cells (ca. 470 nm)^23^. The red-shifted absorption maximum (ca. 460 nm) in comparison with retinal alone (ca. 360 nm) indicates that ACR2 has the protonated retinal Schiff base. Thus, ACR2 is expressed in *E. coli* cells not only as an apoprotein but also as a holoprotein with a retinal chromophore. Then, to identify the substrate ion(s) of ACR2, we performed similar experiments in solutions containing different salts, NaBr, NaI, NaF, NaNO_3_ or Na_2_SO_4_, where the ionic strength was kept constant. Figure 2c shows the light-induced pH change of cells expressing ACR2 in the presence of 10 μM CCCP. The signal almost disappeared in the solution containing Na_2_SO_4_, suggesting that Na^+^ and SO_4_^2−^ are not substrate ions, while a significant and strong signal was observed in the solution containing NaBr, suggesting that Br^−^ can be a substrate ion as well as Cl^−^. More than 3 independent experiments were performed and the averaged relative initial slope was obtained, as shown in Fig. 2d. From these data, we concluded that ACR2 transports monovalent anions including Cl^−^ and Br^−^. These results are generally consistent with the first report for ACR2, in which ACR2 transported anions, including Cl^−^ and Br^−^ but not SO_4_^2−^, by a light-gated anion channel activity, when expressed in HEK293 cells^23^. Thus we demonstrate here the first functional expression of a eukaryotic ChR-like protein in *E. coli* cells.

### Inhibitory role of R84 in ACR2 on its Cl^−^ transport activity

**Figure 3:**
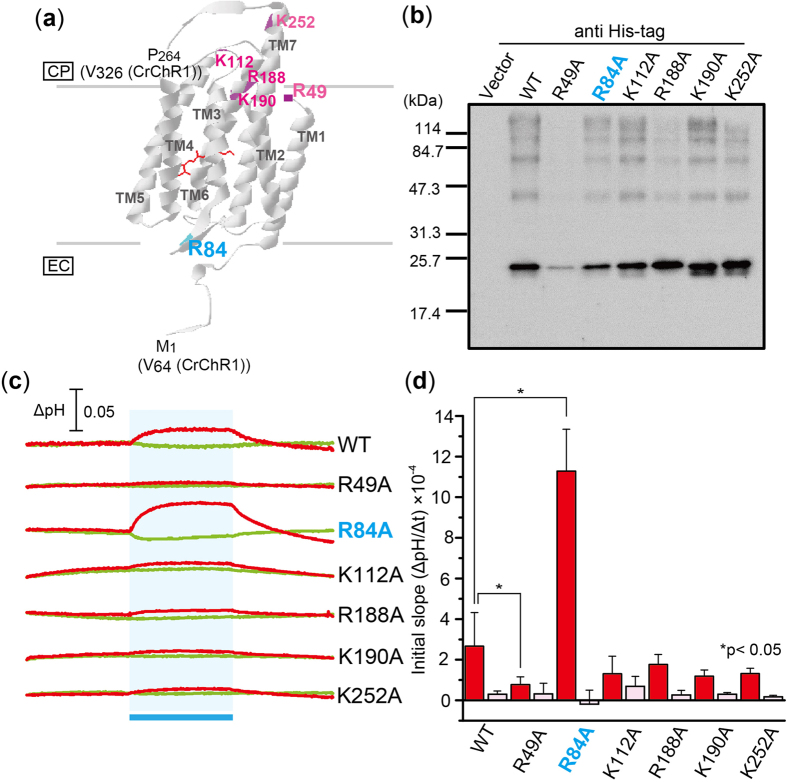
Effects of mutations on conserved basic amino acid residues in ACR2. (**a**) Locations of mutations. The conserved basic amino acid residues in ACR2 are mapped on the crystal structure of a chimeric ChR (C1C2) from *C. reinhardtii* (PDB: 3UG9)^21^. CP and EC indicate the cytoplasmic and extracellular side, respectively. (**b**) Detection of protein expression of wild-type ACR2 and its mutants. These proteins were detected using an anti His-tag antibody. Cells harboring the pET22b vector plasmid alone were used as a negative control. (**c**) Light-induced pH changes of *E. coli* cells expressing ACR2 mutants in a solution containing 300 mM NaCl. The cell suspension was illuminated with blue light (480 ± 10 nm, ca. 9 mW/cm^2^) for 3 min (blue stripe). The red and green lines indicate pH changes in the presence or absence of 10 μM CCCP, respectively. (**d**) Comparison of the ion transport activity of the mutant proteins. The initial slope amplitudes of the light-induced pH changes of *E. coli* cell suspensions in 300 mM NaCl (red) or 100 mM Na_2_SO_4_ (pink) are plotted. Several independent experiments were averaged (n = 3–18). The error bars represent the standard deviation.

The *E. coli* expression system for ACR2 established in this study dramatically improves the expression speed and makes it simple, faster, and cheaper to prepare mutant proteins. By utilizing the *E. coli* expression system, we then tried to identify residues which play important roles in the anion (Cl^−^) transport activity. In this study, to investigate the characteristics of ACRs, we focused on the conserved residues in ACRs (ACR1, ACR2, and Gt161302), which are not conserved in the other microbial rhodopsins. Because ACR2 is an anion transporter, here we focused on 6 basic amino acid residues, R49, R84, K112, R188, K190 and K252 (Fig. 3a), which are completely conserved among ACRs (Figure S1). R90 in ACR2 is completely conserved not only in ACRs, but also in all of microbial rhodopsins (Figure S1). Therefore, R90 is excluded from the mutagenesis. On the basis of the crystal structure of a chimeric ChR^21^, only R84 is assumed to be located at the extracellular side (Fig. 3a). Figure 3b shows western blot analysis of the wild-type ACR2 (WT) and its mutants. Except for R49A, the mutant proteins were successfully expressed in *E. coli* cells as like as the wild-type ACR2. We then measured light-induced pH changes of *E. coli* cells expressing the mutant proteins in a solution containing 300 mM NaCl in the presence (red lines) or absence (green lines) of CCCP (Fig. 3c). The initial slope amplitudes of the light-induced pH change from 0 to 10 sec upon illumination (see Fig. 1c) were obtained in more than 3 independent experiments (n = 3–18). Many of the mutant proteins (K112A, R188A, K190A and K252A) exhibited similar ion transport activities to the wild-type ACR2 (red bars in Fig. 3d), while one of them, R49A, significantly reduced the signal amplitude, suggesting that R49 is important for the protein expression. Of note, the pH change signal was greatly enhanced in R84A (ca. 4-fold) in comparison with the wild-type (red bars in Fig. 3d), indicating the inhibitory role of R84. These pH changes in the presence of CCCP are greatly impaired by addition of SO_4_^2−^ (pink bars in Fig. 3d), suggesting that these mutants don’t alter the ion selectivity.

**Figure 4:**
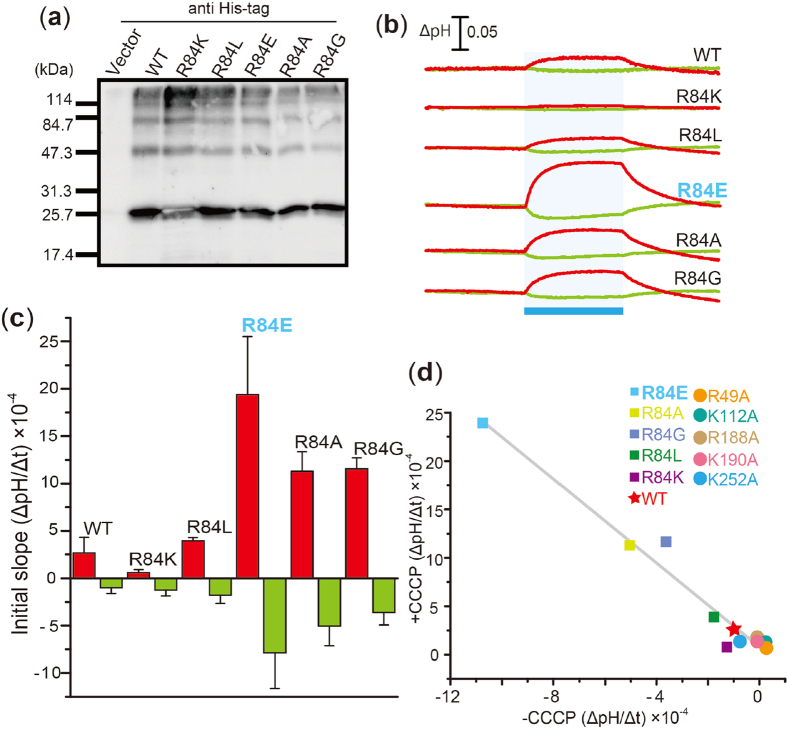
Anion transport activity in R84 mutants. (**a**) Detection of protein expression of R84 mutants. The mutant proteins were expressed in *E. coli* cells with a His-tag at the C-terminus. These proteins were detected using an anti His-tag antibody. Cells harboring the pET22b vector plasmid were used as a negative control. (**b**) Light-induced pH changes of *E. coli* cell suspensions expressing R84 mutant proteins in a solution containing 300 mM NaCl. The suspensions were illuminated with blue light (480 ± 10 nm, ca. 9 mW/cm^2^) for 3 min (blue stripe). The red and green lines indicate pH changes in the presence or absence of 10 μM CCCP, respectively. (**c**) Comparison of the ion transport activity of the R84 mutant proteins. The initial slope amplitudes of the light-induced pH changes of *E. coli* cell suspensions in 300 mM NaCl are plotted. Several independent experiments were averaged (n = 3–18). (**d**) Correlation between the increases and the decreases in pH upon blue-light illumination. The initial slope amplitudes of cells expressing ACR2 are plotted. The correlation coefficient was determined as 0.96.

To investigate whether charge and/or size is important for the inhibitory role of R84 on ion transportation, we replaced R84 with other residues, Lys (R84K), Leu (R84L), Glu (R84E) and Gly (R84G), as shown in Fig. 4. The mutant proteins were constructed to change the side chain volumes in which the order of the side chain volumes is known to be K > L > E > A > G, and two of the mutants, R84K and R84E, were constructed to investigate the role of the positive charge. Western blot analysis revealed that the mutant proteins were successfully expressed in *E. coli* cells as like as the wild-type ACR2 (Fig. 4a). Only for R84K, the band was visualized as white one. Therefore we confirmed by further experiments that the expression level of R84K is almost comparable to that of WT (see supplementary Figure S2).

We then measured the light-induced pH change of cells expressing the mutant proteins in the presence (red lines) or absence (green lines) of 10 μM CCCP (Fig. 4b). Noteworthy, 3 of the mutant proteins, R84E, R84A and R84G, exhibited relatively large increases and decreases in pH corresponding to inward Cl^−^ and outward H^+^ movement, respectively (Fig. 4b and c). On the other hand, R84K was less effective than the wild-type ACR2 (i.e., R84). Two of the variants with a positive charge at the position 84 (i.e., WT and R84K) showed weak ion transport activities, while a variant with a negative charge at the position 84 (i.e., R84E) showed strong ion transport activity (Fig. 4b and c). The activities of the variants with a neutral side chain (i.e., R84L, R84A and R84G) are located between them (Fig. 4b and c). From these results, we assumed that the positive charge at position 84 is critical for the inhibition of light-dependent Cl^−^ transportation. The initial slope amplitudes of the wild-type ACR2 and its mutants constructed in this study were plotted as shown in Fig. 4d. The good correlation indicates that Cl^−^ transportation is tightly coupled with simultaneous H^+^ transportation. Although the origin of the light-induced decrease in pH without CCCP is unclear, these results arise a possibility that ACR2 works as a light-dependent Cl^−^/H^+^ antiporter, when it is expressed in *E. coli* cells.

**Figure 5:**
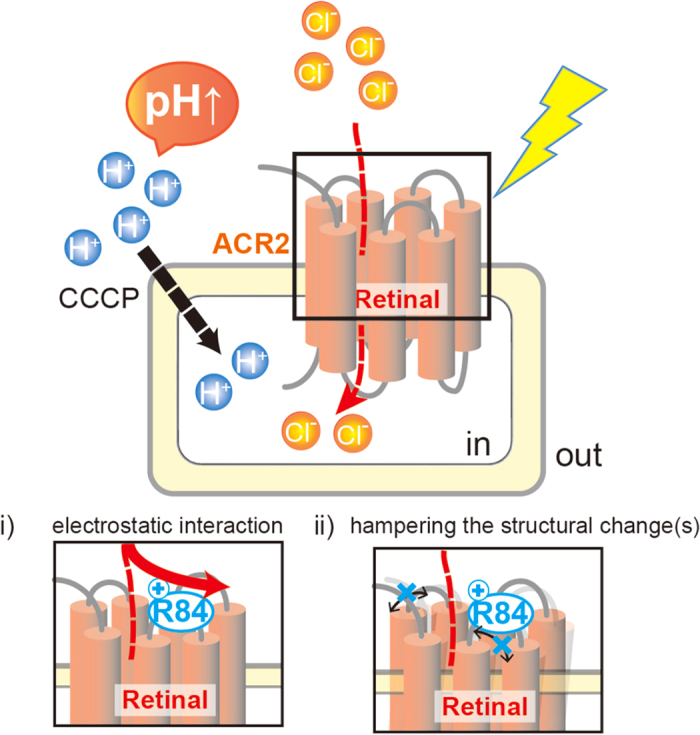
Model for the light-dependent Cl^−^ transportation of ACR2. The light-induced inward secondary H^+^ movement across the membrane by the inward Cl^−^ channeling activity of ACR2 is facilitated by the addition of the protonophore CCCP, resulting in the increase in pH on the outside of the cells. The basic residue R84 behaves as an inhibitor during the anion transportation. Two possibilities for the inhibition are proposed as i) and ii).

Regarding the molecular mechanism of Cl^−^ transportation, in this study, we demonstrated the inhibitory role of the positive charge of R84 on inward Cl^−^ transportation. Of note, R84 is conserved in ACRs but not in other microbial rhodopsins (Figure S1). From the crystal structure of a chimeric ChR named C1C2^21^, R84 is estimated to be located on a loop (B-C loop) between TM helix-2 (TM2) and TM helix-3 (TM3). In the structure of C1C2, 4 TM helices, TM1, TM2, TM3 and TM7, consist of the putative cation transport pathway. From that background, we hypothesize two possibilities for the inhibitory role of R84 in Cl^−^ transportation: (i) R84 captures a Cl^−^ by electrostatic interaction at the entrance of the anion conduction pathway, or (ii) the positive charge of R84 hampers the structural change(s) important for anion transportation (Fig. 5). Thus it is quite reasonable to assume that the mutations directly affect ion transport activity. However, in the strict sense, there are three alternative possibilities for the differences in intensity of the pH change in the mutants as follows: (i) differences in expression level of the holoprotein, but not the apoprotein; (ii) differences in the incorporation efficiency of the retinal and (iii) differences in the absorption maximum. To investigate them spectroscopically, we need to purify the proteins. However we found that the ACR2 proteins including the mutants are rapidly denatured in the detergent (DDM) micelles. The instability made it difficult to purify the proteins and to confirm the three alternative possibilities. Thus, for further quantitative analysis, the purification of ACR2 and its spectroscopic characterization are needed and they are our next focus.

## Conclusion

In this study, we developed a functional expression system for ACR2 in *E. coli* cells. Utilizing that system, we demonstrated that the positive charge of R84 on the B-C loop inhibits its anion transport activity. The enhancement of the anion transport activity of R84E would make ACR2 more effective optogenetic neural silencer.

## Methods

### Gene preparation and protein expression in *E. coli*

The gene encoding the 7 TM domains of ACR2 (GenBank ID: KP171709.1, amino acid residues from the first to the 266th position) was attached with NdeI and XhoI restriction enzyme sites and was chemically synthesized by GenScript Japan Inc. (Tokyo, Japan). The codons were optimized for *E. coli* codon usage and the codon-optimized gene was inserted into the pET22b plasmid vector (Novagen, Madison, WI). Consequently, the plasmid encodes the 7TM domain with a His_6_-tag at the C-terminus (Figure S1). The mutant genes were constructed using the QuikChange site-directed mutagenesis method (Agilent Technologies, Inc., Santa Clara, CA) as described previously^24^. The constructed plasmids were analyzed using an automated sequencer (ABI 3500 Genetic Analyzer, Life Technologies Ltd., Carlsbad, CA) to confirm the expected nucleotide sequences. The *E. coli* DH5α strain was used as a host for DNA manipulation.

Proteins were expressed in *E. coli* BL21(DE3) cells using essentially the same method described previously^25^. Briefly, freshly colonized cells harboring the expression plasmids were inoculated in 100 mL LB medium containing 50 μg/mL ampicillin and were grown at 30 °C. After 6 hr of preculture, the medium was transferred to 1.9 L LB medium containing 50 μg/mL ampicillin. The cells were grown at 30 °C until the optical density at 660 nm reached 0.2–0.4. The cells were then kept on ice for 15 min. Isopropyl β-D-thiogalactopyranoside (IPTG, final concentration = 0.5 mM) (Wako Pure Chemical Industries, Ltd., Osaka, Japan) and all-*trans* retinal (final concentration = 10 μM) (Sigma-Aldrich, St. Louis, MO) were then added to the culture medium and were incubated at 18 °C for 12–14 hr to induce protein expression. After that, the cells were harvested by centrifugation (7,500× g for 10 min at 4 °C).

### SDS-PAGE and western blot analysis

SDS-PAGE and western blot analysis were carried out to confirm the apoprotein expression according to the standard method as described previously^25^. Briefly, *E. coli* cells being induced to express the wild-type ACR2 and its mutants were suspended in a buffer containing 20 mM Tris-HCl (pH 8.0) and 100 mM NaCl. The optical density at 660 nm of all samples was initially set at 0.5. The cell suspensions were then diluted 10 times with the same buffer. The diluted samples were suspended in the standard SDS-PAGE loading buffer containing 5% 2-mercaptoethanol, were heated at 95 °C for 5 min and then centrifuged to remove impurities (20,000× g for 2 min at 25 °C). After that, the samples were subjected to 12% acrylamide SDS-PAGE. Immunoblotting analysis was then performed using an anti His-tag HRP conjugate antibody (Novagen) by the standard method. *E. coli* cells harboring the pET22b vector plasmid alone were simultaneously analyzed as a negative control using the same experimental procedures.

### Measurements of ion transport activity

The ion transport activity was measured by light-induced pH changes using essentially the same apparatus and method as described previously^22^. Briefly, *E. coli* BL21(DE3) cells harboring plasmids were suspended and washed three times in solutions containing 300 mM NaCl, NaBr, NaI, NaF, NaNO_3_ or 100 mM Na_2_SO_4_, to remove LB culture medium. After that, cells were resuspended in the same solutions, and kept on ice for 1 hr, after which they were used for measurements. The samples were kept in the dark until the pH of the solution became stable, and then were illuminated using a Xe lamp through a series of band-pass filters (400 ± 10 nm, 418.5 ± 9 nm, 450 ± 10 nm, 480 ± 10 nm, 500 ± 10 nm, 520 ± 10 nm and 550 ± 10 nm). The light power was measured and adjusted to be 8–10 mW/cm^2^ using an optical power meter (#3664, Hioki, Ueda, Japan) with an optical sensor (#9742, Hioki, Ueda, Japan). If necessary, the protonophore carbonyl cyanide *m*-chlorophenylhydrazone (CCCP, Sigma-Aldrich) was added to a final concentration of 10 μM. The temperature was maintained at 25 °C. Data for each action spectrum were fitted by a normal Gaussian distribution. Statistical analyses were performed using GraphPad Prism 6.07 software. Statistical significance was evaluated by the Mann-Whitney test for two group comparisons.

## Additional Information

**How to cite this article**: Doi, S. *et al*. An inhibitory role of Arg-84 in anion channelrhodopsin-2 expressed in *Escherichia coli. Sci. Rep.* **7**, 41879; doi: 10.1038/srep41879 (2017).

**Publisher's note:** Springer Nature remains neutral with regard to jurisdictional claims in published maps and institutional affiliations.

## Supplementary Material

Supplementary Information
