# Supplementary material for: An inhibitory role of Arg-84 in anion channelrhodopsin-2 expressed in Escherichia coli
Source: Sci Rep. 2017 Feb 2;7:41879. doi: 10.1038/srep41879 (PMC5288786; doi:10.1038/srep41879)
Supplement: Supplementary Information [file srep41879-s1.pdf]

An inhibitory role of Arg-84 in anion channelrhodopsin-2 expressed in *Escherichia coli*

Satoko Doi[1], Takashi Tsukamoto[1], Susumu Yoshizawa[2], & Yuki Sudo[1,*]

[1]Graduate School of Medicine, Dentistry and Pharmaceutical Sciences, Okayama University, Okayama 700-8530, Japan.

[2]Atmosphere and Ocean Research Institute, The University of Tokyo, Chiba 277-8564 Japan.

*To whom correspondence should be addressed: Yuki Sudo; Telephone: +81-86-251-7945, E-mail: sudo@okayama-u.ac.jp

SUPPLEMENTARY INFORMATION

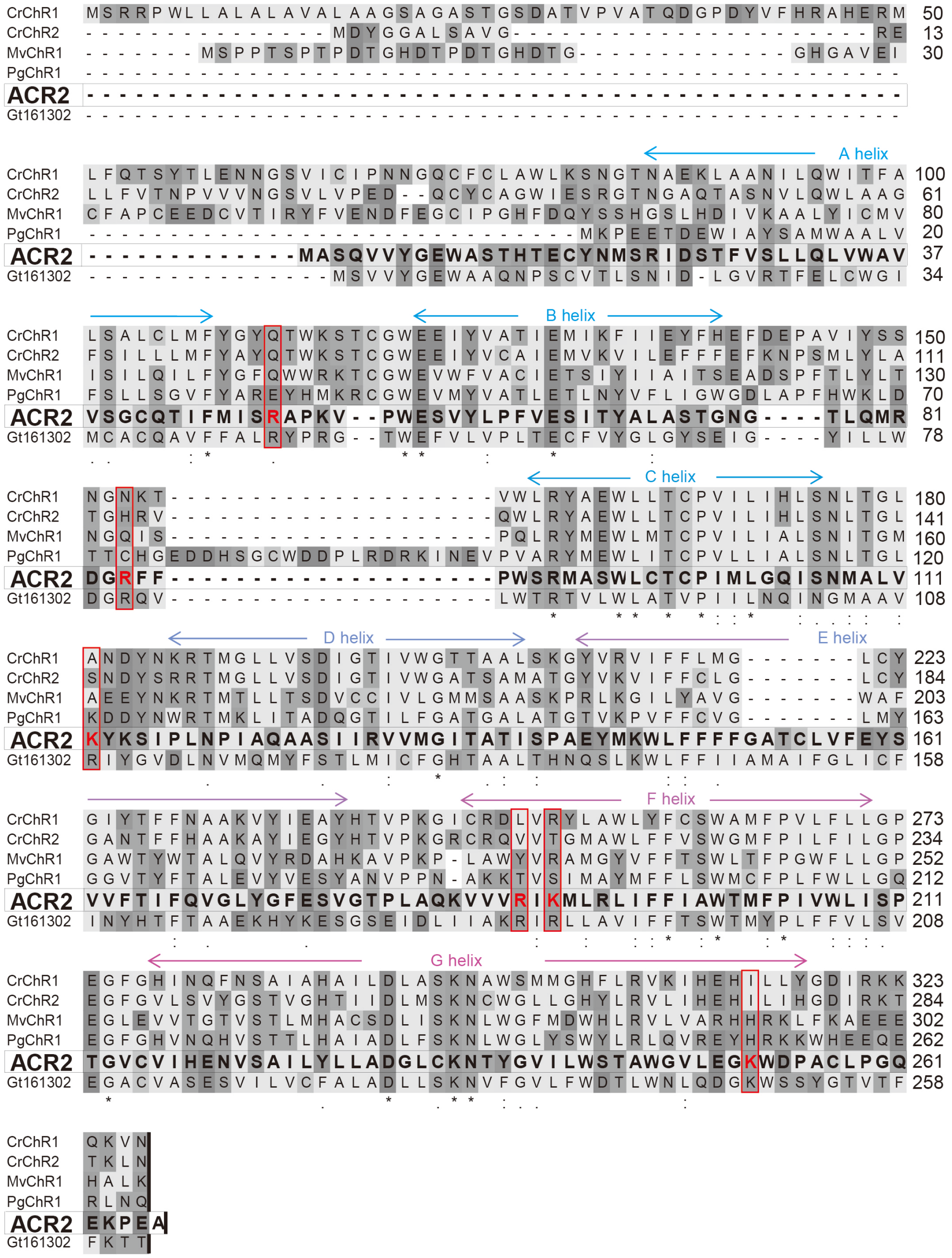

Figure S1. Amino acid sequence alignment of ChR-like proteins.

Seven TM helices are indicated as A, B, C, D, E, F and G. ACR2 from *G. theta* (GtACR2) focused on in this study is highlighted in bold characters and the mutated basic amino acid residues are shown in red.

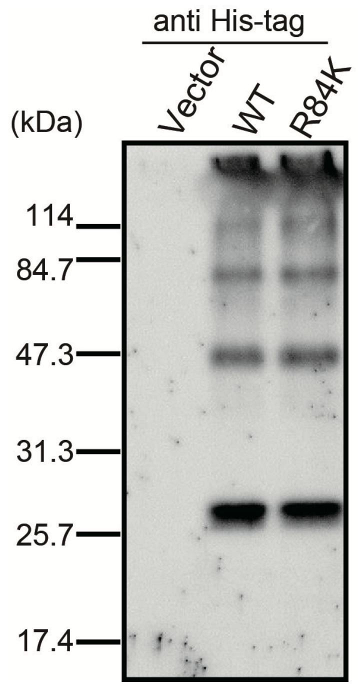

Figure S2. Western blot analysis for wild-type (WT) and R84K mutant of ACR2.

The protein was expressed in *E. coli* BL21(DE3) cells with a His-tag at the C-terminus and was detected using an anti His-tag antibody. Cells harboring the pET22b vector plasmid alone were used as a negative control (vector).
